# Structure and Bioactive Properties of Novel Textile Dyes Synthesised by Fungal Laccase

**DOI:** 10.3390/ijms21062052

**Published:** 2020-03-17

**Authors:** Jolanta Polak, Kamila Wlizło, Rebecca Pogni, Elena Petricci, Marcin Grąz, Katarzyna Szałapata, Monika Osińska-Jaroszuk, Justyna Kapral-Piotrowska, Bożena Pawlikowska-Pawlęga, Anna Jarosz-Wilkołazka

**Affiliations:** 1 Department of Biochemistry and Biotechnology, Institute of Biological Sciences, Maria Curie-Skłodowska University, Akademicka 19, 20-033 Lublin, Poland; kamila.wlizlo@poczta.umcs.lublin.pl (K.W.); graz@poczta.umcs.lublin.pl (M.G.); katarzyna.szalapata@poczta.umcs.lublin.pl (K.S.); monika.osinska-jaroszuk@poczta.umcs.lublin.pl (M.O.-J.); anna.wilkolazka@poczta.umcs.lublin.pl (A.J.-W.); 2 Department of Biotechnology, Chemistry and Pharmacy, University of Siena, Via A. Moro 2; 53100 Siena, Italy; rebecca.pogni@unisi.it (R.P.); elena.petricci@unisi.it (E.P.); 3 Department of Functional Anatomy and Cytobiology, Institute of Biological Sciences, Maria Curie-Skłodowska University, Akademicka 19, 20-033 Lublin, Poland; justyna.kapral-piotrowska@poczta.umcs.lublin.pl (J.K.-P.); bozena.pawlikowska-pawlega@poczta.umcs.lublin.pl (B.P.-P.)

**Keywords:** biotransformation, laccase, phenazine, bioactive dyes, antimicrobial, antioxidative compounds

## Abstract

Novel sustainable processes involving oxidative enzymatic catalysts are considered as an alternative for classical organic chemistry. The unique physicochemical and bioactive properties of novel bio-products can be obtained using fungal laccase as catalyst. Among them are textile biodyes synthesised during oxidation of substrates belonging to the amine and methoxy organic derivatives. The process of synthesis occurs in mild conditions of pH, temperature, and pressure, and without using harmful oxidants. The effect of fungal laccase activity on the substrates mixture transformation efficiency was analysed in terms of antimicrobial dye synthesis on a large scale. Three new phenazine dyes, obtained in the presence of laccase from *Cerrena unicolor*, were studied for their structure and properties. The phenazine core structure of the products was a result of tri-molecular transformation of aminomethoxybenzoic acid and aminonaphthalene sulfonic acid isomers. One of the compounds from the synthesised dye, namely 10-((2-carboxy-6-methoxyphenyl)amino)-11-methoxybenzo[a]phenazine-8-carboxylic acid, was able to inhibit the growth of *Staphylococcus aureus*. The high concentration of substrates (5 g/L) was efficiently transformed during 72 h in the mild conditions of pH 4 with the use of laccase with an activity of 200 U per g of the substrates mixture. The new bioactive dye exhibited excellent dyeing properties with concomitant antibacterial and antioxidative activity. The proposed enzyme-mediated synthesis represents an alternative eco-friendly route for the synthesis of novel antimicrobial compounds with high importance for the medical textile industry.

## 1. Introduction

Laccases, especially those of fungal origin, are universal, cheap, and easy to handle biocatalysts with several applications [1,2]. The synthesis of the long list of novel organic compounds, e.g., substituted imidazoles, cyclohexenes, phenoxazines, or azo dyes, is possible in one-pot reactions via laccase-mediated homo- or heteromolecular transformations of different substrates belonging to the amino-, methoxy-, phenoxy-benzene or -naphthalene derivatives [3]. The compounds mentioned above, especially those belonging to phenazines or phenoxazines, are very promising due to their possible bioactivity and dyeing properties. In nature, phenazines with broad bioactivity are products of the idiophase of different bacteria, especially from *Pseudomonas* and *Streptomyces* strains [4,5]. Both natural and synthetic phenoxazines and phenazines known from the literature exhibit biological activities such as antibacterial [6,7,8], antitumoral [9], antiproliferative [10], and antioxidant [11] properties. The chemical route of phenazine synthesis requires the use of coupling agents and additives such as benzene, BBr_3_, DMF, and various other harsh, toxic, and mutagenic compounds [12]. In contrast to the chemical synthesis, phenoxazine and phenazine compounds can be easily obtained through the biocatalysis due to the action of fungal or bacterial laccase and the use of aminophenolic precursors [13,14,15,16]. Phenoxazines can be synthesised in laccase-mediated homomolecular coupling reactions of different ortho-aminophenols or ortho-aminonaphtalenes [13,14], whereas phenazines can be obtained through the heteromolecular transformation of phenol derivatives with aromatic amines [15,17]. The synthesis of new substances with the phenazine core is highly desirable, as it may result in the discovery of new chemicals with new properties, including bioactivity. Herein, we report the structure of three different phenazines obtained through the laccase-mediated heteromolecular transformation of aminomethoxybenzoic acid with three isomers of aminonaphthalene sulfonic acid. Red-coloured products were obtained and one of them was found to exert an antibacterial effect against a *Staphylococcus aureus* strain. All these processes are very promising alternatives to classical organic synthesis having many disadvantages, such as the use of harsh reaction conditions as well as harmful and toxic organic additives. In contrast, during the laccase-mediated synthesis of novel organic compounds, the main co-substrate is oxygen and the only waste by-product is water, which renders this process “green”.

The aim of this study was to synthesise red-coloured dyes, displaying dyeing, antioxidative, and even antimicrobial properties, in the fungal laccase-mediated eco-friendly process. The process optimisation included the selection of (1) an optimal value of pH, (2) concentrations of substrates and their molar ratios, and (3) biocatalyst activity as the key parameters for the enzymatic process implementation in the industry. To our knowledge, this is the first “de novo” synthesised compound with both antioxidative and antimicrobial properties obtained by the use of fungal laccase as the catalyst. From this point of view, fungal laccase can be considered as a useful biocatalyst for the synthesis of bioactive dyes.

## 2. Results

### 2.1. Electrochemical and Kinetic Parameters of Substrates and Their Reactivity during Oxidation with Fungal Laccase

**Figure 1:**
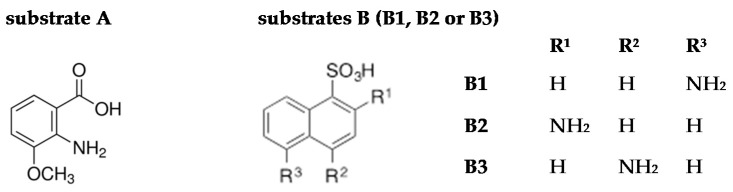
Structure of substrates used for laccase (LAC)-mediated heteromolecular transformations. R—the designation of specific substituents hydrogen atom (H) or amine group (NH_2_) in individual places.

Four different substrates belonging to benzene (substrate A) and naphthalene derivatives (substrates B1, B2, and B3) were analysed as potential substrates for LAC-mediated oxidation (Figure 1).

Three different parameters, i.e., the electrochemical potential, oxygen demand, and kinetic constant *K*_M_ were analysed as key factors playing an important role during the enzymatic oxidation of different substrates to reactive radicals. Substrate A belonging to the carboxylic acid and possessing an amine and methoxy group at position 2 and 3, respectively, has both the lowest oxidation potential and the lowest value of *K*_M_ with simultaneously the highest oxygen demand observed during its oxidation by LAC (Table 1).

In contrast to the 2-amino-3-methoxycarboxylic acid (substrate A), aminonaphthalene derivatives (substrates B1, B2, and B3) were much more slowly oxidised by LAC during the homomolecular reaction. The lack of additional reactive substituents, especially phenolic groups, resulted in an increase in the oxidation potential value and the *K*_M_ value, with a simultaneous decrease in oxygen uptake (Table 1). Moreover, the values of these parameters largely depend on the position of the amino group attached to the naphthalene core. Substrate B2, having the amino group in the ortho position in respect to the sulfonic group, was not effectively oxidised by LAC (*K*_M_ = 17 mM) due to the high value of the oxidation potential. The localisation of the amino group at positions 4 and 5 of substrates B3 and B1, respectively, in respect to the sulfonic group of naphthalene, led to the reduction of the oxidation potential and *K*_M_ values. Consequently, the possible oxidation of these substrates by LAC is confirmed by the lower value of *K*_M_ corresponding to 5.38 mM and 3.94 mM, respectively (Table 1).

### 2.2. Heteromolecular Transformation of Substrates

**Figure 2:**
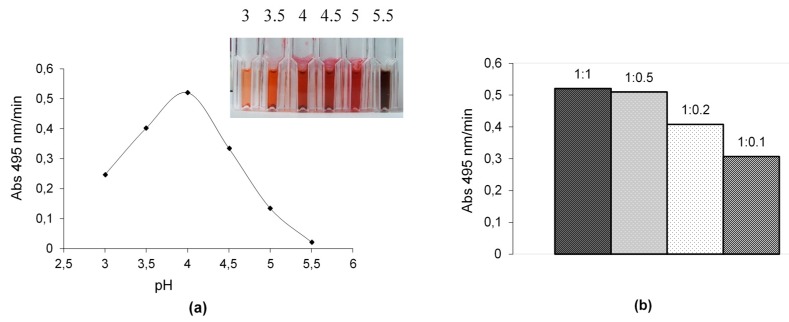
Influence of the pH value (**a**) and the molar ratio of substrates A and B2 (1:1, 1:0.5, 1:0.2, 1:0.1—A:B2 molar ratio) (**b**) on the rate of phenazine AB2 synthesis mediated by LAC.

Products obtained from heteromolecular transformation of substrate A with one from the substrate B were intensively coloured and exhibit maximum absorption around 495 nm for compounds AB1 and AB2 and at 505 nm for compound AB3 at optimal pH of 4.5, 4 and 4.5 respectively, and therefore can be considered as textile dyes. Moreover, different colouration of the end-product was observed at pH 5.5, which confirmed the optimal value of pH in the range from 4 to 4.5 (Figure 2a).

No differences in the spectrum and TLC chromatograms of the products obtained were noted in the presence of 10% of the organic solvents. Therefore, the optimal transformation mixture did not contain these solvents, which confirms the “green and more ecological character” of the transformation process.

In the optimal buffer conditions, the effect of a different molar ratio of A and B on the rate of transformation into a novel product was investigated. The best rate of biotransformation was obtained during oxidation of the equimolar ratio of substrate A with substrate B2 (Figure 2b) as well as substrate A with substrates B3 or B1. In such conditions, the isolated yields of the main products AB1, AB2, and AB3 estimated from the actual and theoretical yield and based on stoichiometric calculations were in the range from 19 to 27%, depending on the product obtained (Table 2).

**Figure 3:**
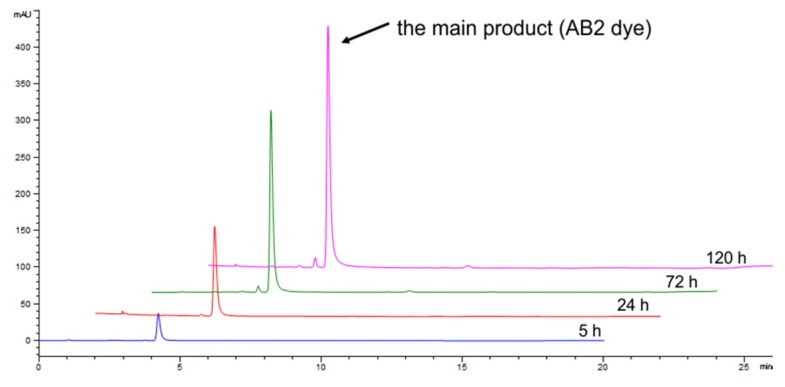
HPLC chromatograms of the transformation mixture detected at 500 nm containing the main product of the AB2 dye (absorption units, mAU) synthesised after 5, 24, 72, and 120 h of 5 g/L mixture of A and B2 substrates (methanol fraction).

Based on the HPLC analysis of the transformation mixture (monitored at 500 nm), it can be concluded that low yield of the main dye is due to the presence of other different orange-coloured products arising during the transformation, with lower intensity, which have not been isolated and analysed to assess their molecular weight as well as bioactive properties (Figure 3).

**Figure 4:**
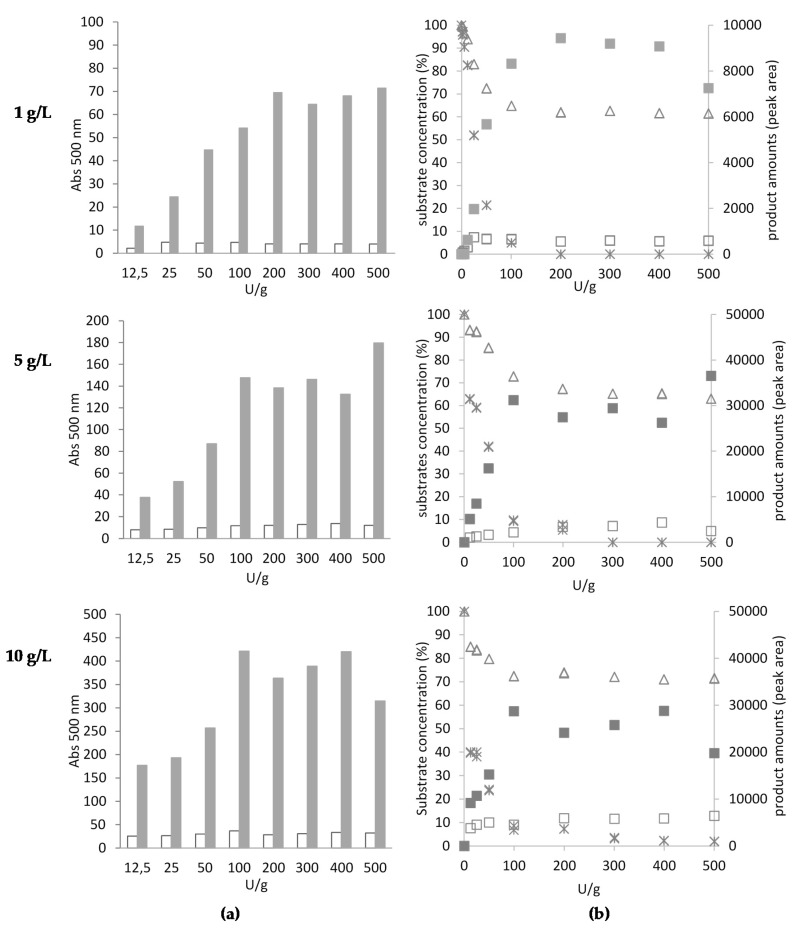
Effect of LAC activity (U/g) on the amount of AB2 dye expressed as the absorbance at 500 nm, dissolved in methanol (filled bars) and water (empty bars) fractions (**a**) and the peak area obtained from HPLC analysis (**b**) of methanol (filled squares) and water (empty squares) fractions of products after 48-h oxidation. Residual concentrations of A and B2 substrates (%) were analysed using HPLC; substrates are marked as follows: substrate A residual concentration (%)—cross; substrate B2 residual concentration (%)—triangle.

The effect of LAC activity ranging from 12.5 to 500 U/g was checked in terms of the potential upscaling of the AB2 dye synthesis process. LAC was also used for the transformation of higher concentrations of the substrates (1, 5, and 10 g/L) within 48 h. Based on the value of dye absorbance (at 500 nm) and the main AB2 compound peak area, the optimal LAC activity was around 200 U/g for all tested concentrations of substrates A and B2 (Figure 4).

**Figure 5:**
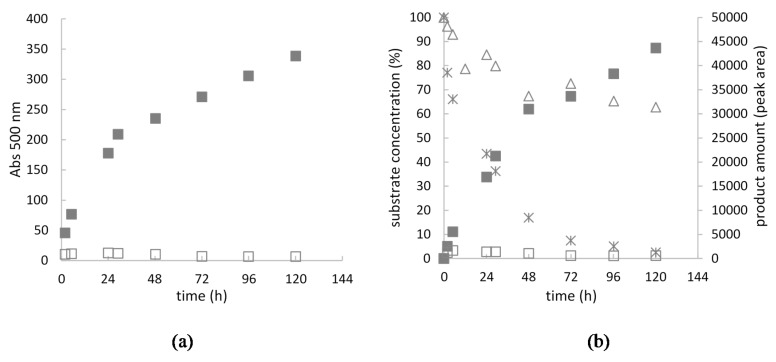
Amount of AB2 dye obtained from substrate A and B2 equimolar oxidation (total concentration of mixture 5 g/L) using fungal LAC (200 U/g) during 5 days of transformation. The amount of the dye was expressed as total absorbance at 500 nm (**a**) and AB2 dye peak area obtained from HPLC analysis (**b**) of water and methanol fractions. Residual concentration of A and B2 substrates (%) was analysed using HPLC. Substrates and products are marked as follows: AB2 dye fraction dissolved in water–empty square; AB2 dye fraction dissolved in methanol–filled square; substrate A residual concentration (%)–cross; substrate B2 residual concentration (%)–triangle.

The detailed analysis of the transformation of 5 g/L of the A and B2 mixture was performed for 5 days using LAC with the final activity of 200 U per gram of substrate (Figure 5). Based on the HPLC analysis, it can be concluded that the nearly total transformation of substrate A occurred within 72 h (Figure 5).

After this time, an increase in the amount of the product was still observed (both absorbance and peak area of the main compound of the AB2 dye, which was probably caused by non-enzymatic coupling reaction of intermediates.

### 2.3. Characteristics of Products Obtained

#### 2.3.1. Structure Analysis

**Figure 6:**
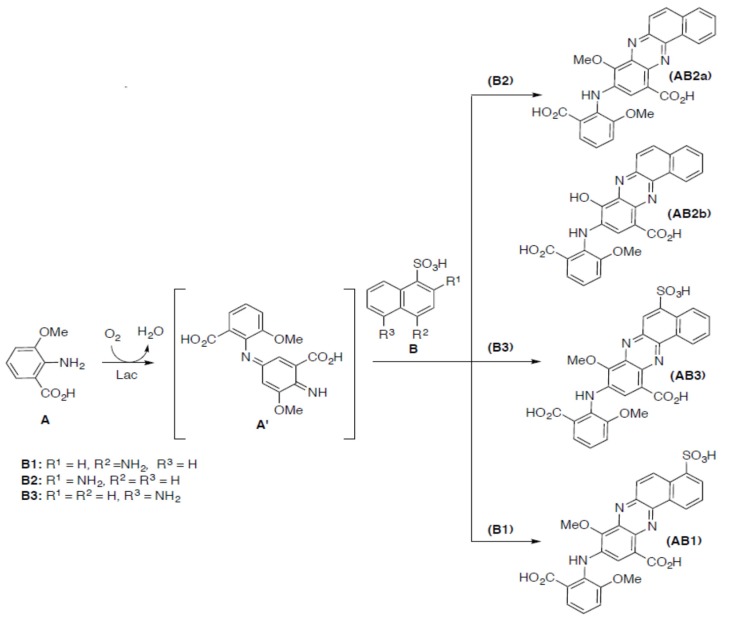
Proposed pathway of synthesis of phenazines (AB1, AB2a, AB2b, and AB3) as a result of LAC-mediated heteromolecular coupling reactions of aminomethoxycarboxylic acid (A) with three different isomers of aminonaphthalene (B1, B2, and B3). Acronyms of substrates and products have been bolded.

Products obtained during the cross-coupling reaction between the equimolar concentrations of substrate A and substrates B1, B2, or B3, namely AB1, AB2, and AB3, respectively, were purified by silica gel flash chromatography and characterised by IR, NMR (Table S1), and UV-Vis spectroscopy and mass spectrometry. As shown in Figure 5, the oxidation of the substrates probably led to the formation of substituted phenazines with a “head to head” framework (Figure 6).

Depending on the substrate B isomer used for the heteromolecular oxidation in the presence of substrate A, four different trimolecular compounds with different solubility in water were obtained during the reaction (Figure 6). The kinetic parameters corroborate the structure of the phenazines obtained. In the case of the B2 isomer, two different products, namely AB2a (10-((2-carboxy-6-methoxyphenyl)amino)-11-methoxybenzo[a]phenazine-8-carboxylic acid) and AB2b (10-((2-carboxy-6-methoxy-phenyl)amino)-11-hydroxybenzo[a]phenazine-8-carboxylic acid), were detected during the HPLC-MS analysis. The ES mass analysis of AB2a and AB2b showed peaks at *m*/*z* 456 and 470 [M+H]^+^, respectively, in positive mode and peaks at *m*/*z* 454 and 468 [M-H]^−^, respectively, in negative mode. The ES mass spectra of the AB1 product (10-((2-carboxy-6-methoxyphenyl)amino)-11-methoxy-4-sulfobenzo[a]phenazine-8-carboxylic acid), and the AB3 product (10-((2-carboxy-6-methoxyphenyl)amino)-11-methoxy-5-sulfobenzo[a]phe-nazine-8-carboxylic acid) showed the peaks at *m*/*z* 550 in positive mode and at *m*/*z* 548 [M-H]^−^ in negative mode.

#### 2.3.2. Antioxidative Properties of Phenazine Dyes

The antioxidative properties of phenazines were evaluated using a chemiluminescence method due to the intensive red colouration of dyes, which can interfere with the DPPH or ABTS assays, and therefore they are not useful for these purposes. All the tested dyes exhibited antioxidative potential with an EC_50_ value in the range from 0.44 to 0.63 mg per mL (Table 3).

#### 2.3.3. Antimicrobial Properties of Phenazine Dyes

**Figure 7:**
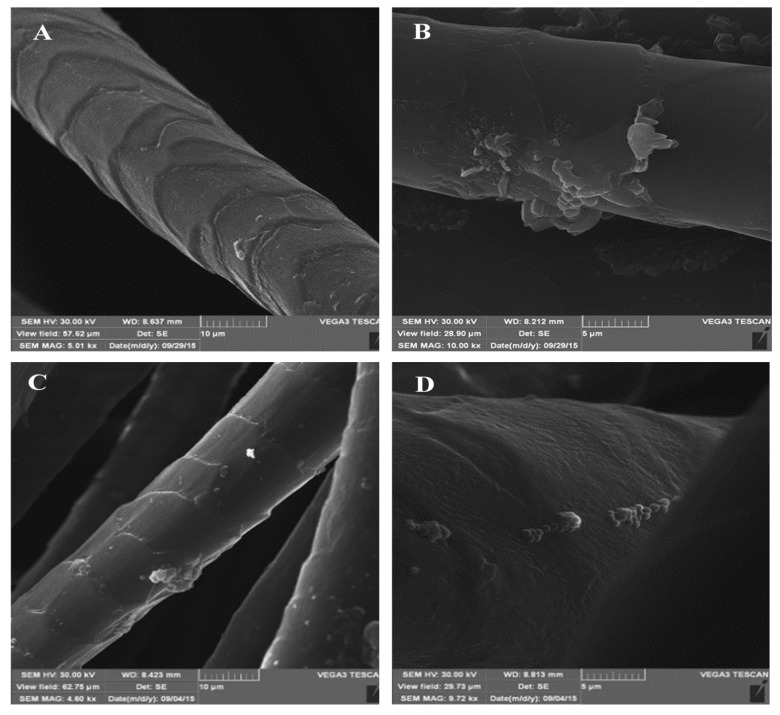
SEM pictures of wool fabrics dyed with 10% AB2 dye (**A** and **B**) and corresponding control samples of non-dyed wool fabrics (**C** and **D**) after 24-h incubation with the *S. aureus* strain.

All phenazines were tested for their antimicrobial effect against *E. coli* and *S. aureus* strains using qualitative (agar diffusion test) and quantitative methods. In the case of the AB2 compound, it was tested as a mixture of AB2a and AB2b products. From all the tested compounds, AB2 showed inhibition potential against *S. aureus* with a MIC value of 0.3 mg/mL. Simultaneously, no antibacterial effect of the two other tested products, namely AB3 and AB1, was noted in the case of both the *S. aureus* and *E. coli* bacterial strains. Afterwards, the coloured AB2 product was applied for dyeing of wool fabric using 5% and 10% concentrations relative to the mass fibre (omf). The dyed wool fabrics were incubated in the presence of the *S. aureus* strain. After overnight incubation, inhibition of bacterial growth was possible to observe. The application of 10% of the AB2 dye caused nearly 100% growth inhibition of the *S. aureus* strain, whereas nearly 40% growth inhibition was noted at the concentration of 5%. The antibacterial effect of the wool samples dyed with 10% AB2 was confirmed using the scanning electron microscopy (SEM). No bacterial colonies on the dyed wool fabrics were observed (Figure 7A,B) in contrast to the control non-dyed wool samples that contained visible single bacterial cells and colonies (Figure 7C,D).

#### 2.3.4. Dying Properties of AB2 Dye

**Figure 8:**
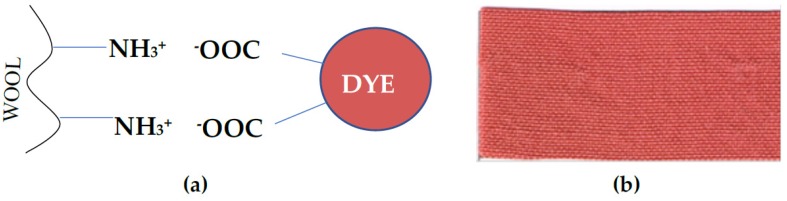
Scheme of dyeing mechanism (**a**) and sample of wool fabric dyed with 1% of AB2 compound (**b**), tested under dyeing quality.

Wool fibres dyed with 1% concentration of AB2 product were reddish coloured (Figure 8b). They were tested for their resistance to physicochemical factors, among them were artificial light, distilled water, washing at 40 °C, alkaline and acidic sweat, and dry and wet rubbing (Table 4).

Tested AB2 dye showed good dyeing quality with moderate resistance in the case of alkaline sweat treatment (Table 4), which indicated the high dyeing potential of the tested compound as fabric dye.

## 3. Discussion

There is a growing need to discover new bioactive compounds due to the increased bacterial resistance to commonly used antibiotics. Literature data have so far described mainly natural dyes and naturally occurring phenols applied as laccase-assisted grafting agents of textile fibres with antimicrobial activity [18,19,20,21]. Moreover, only few papers concern the synthesis of novel LAC-mediated coupling products from phenolics or well-known antibacterial chemicals [3,22,23]. From the broad variety of antimicrobial compounds, phenazines, acting as both electron donors and acceptors, are able to inhibit the growth of bacteria, fungi, parasites, insects, malaria agents, and other organisms; hence, they are promising new bioactive compounds [7,8]. Our results presented in this paper indicate possible laccase-mediated synthesis of new trimolecular phenazines, which exhibit both bioactive and dyeing properties.

Three different mixtures of aminomethoxybenzoic acid and isomers of aminonaphthalenesulfonic acid were transformed by fungal laccase used as the catalyst, into products with very intense red colouration, which has already been mentioned in our previous paper [24]. It is already known, that the type of substituents (e.g., amino, methoxy) has a substantially higher impact on the reactivity of the tested substrates during homomolecular LAC-mediated oxidation than the type of the aromatic ring (benzoic and naphthalene) [25,26]. All of tested substrates possess at least one amino substituent, being a very reactive group for the LAC-mediated coupling reaction with other substrates to yield coloured products [26]. However, only substrate A, containing additional reactive methoxy group, was transformed into orange product about low toxicity during its homomolecular transformation and the dye obtained was able to colour natural fibres [24,25,27]. It was possible due to low values of redox potential and *K*_M_ of substrate A, in contrary to the high values of both redox potential and *K*_M_ of aminonaphthalenesulfonic acids (substrates B). Despite that these three different structural isomers tested as a potential substrate of laccase possess one amino group each (the well-known reactive substituent involving in the LAC-mediated coupling reaction), low intensity coloured product was formed only in the case of substrate B1 homomolecular transformation [27,28]. Different effects of ring activation are observed in different isomers, what in the case of para compounds is responsible for the substrates redox potential lowering and the easier LAC-mediated oxidation [15,28].

**Figure 9:**
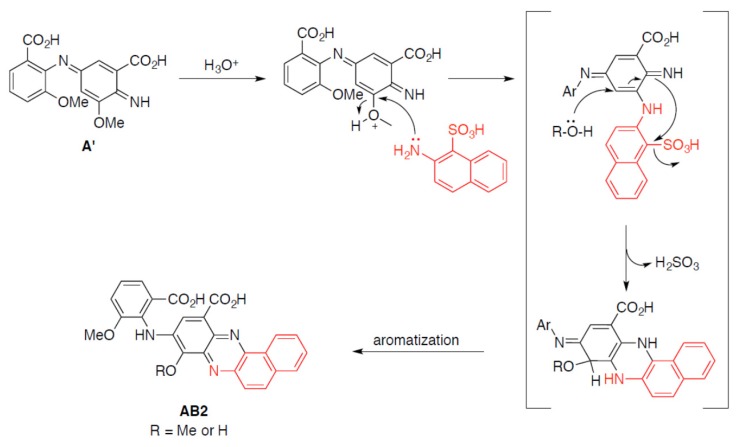
Proposed mechanism of heteromolecular coupling reaction of A’ dimer and B2 substrate (marked in red) during AB2 dye synthesis.

Totally different products were obtained during heteromolecular biotransformation of substrate B isomers in the presence of substrate A, and in these cases, the transformation mixtures were transformed into reddish coloured compounds after few minutes, which indicates the formation of new products [24]. The rate of each reaction at the different pH values exhibited a bell-shaped profile, with the maximal rate at pH 4 in the case of substrates A and B2 mixture (Figure 2a) and at pH 4.5 in the case of substrates A and B3 or substrates A and B1 mixtures (data not shown), which is consistent with the optimum pH profile of fungal laccases [29]. The present results reveal that substrate A, which can be directly oxidised by fungal laccase from the *Cerrena unicolor* strain due to its similar redox potential value, plays a key role in the heteromolecular coupling reaction with all tested aminonaphthalenesulfonic acids [30]. The low redox potential and the low value of *K*_M_ of substrate A suggest a probability of its dimerization into compound A’ as the first step of the LAC-mediated reaction (Figure 6). During this process, the amine radical A formed is involved in the coupling reaction on the para position of a second molecule (A) with the formation of the electrophilic quinondiimine intermediate (A’), which then reacts with a nucleophilic partner (B isomers) followed by intramolecular cyclization and methoxy group rearrangement. The putative reaction mechanism is reported in Figure 9.

Each isomer of substrate B is not readily oxidised by LAC, which is confirmed by its high oxidation potential and high *K*_M_ value. Therefore, it might be a target of a further coupling reaction with the radical form of compound A’ in the second step of the reaction. During this process, a trimolecular phenazine structure is formed. Dye AB2, as a mixture of compounds AB2a and AB2b, exhibited lower solubility in water in contrast to the other products obtained, which can be explained by the lack of the sulfonic group in the structure. In the case of products obtained from the heteromolecular transformation of substrate A with isomers B3 or B1, similar water-soluble phenazines were obtained in both cases. The good water solubility of the AB1 and AB3 products was possible due to the presence of a sulfonic group at positions 4 and 5, respectively.

The high concentration of substrates A and B2 mixture (5 g/L) applied for the transformation, yielded a total consumption of substrate A and nearly 50% consumption of B2 compound after 72 h of synthesis, what confirms our proposed structure of AB2 dye and mechanism of the synthesis. After 72 h of the reaction, an increase in the amount of the product AB2 was still observed (both absorbance and peak area of the main compound of the AB2 dye), which was probably caused by non-enzymatic coupling reaction of intermediates.

The antimicrobial and antioxidative properties of organic compounds very often are associated with the presence of hydroxy substituents acting as electron donors. Tested biodyes possess substituents rich in free electrons, such as methoxy and amine, which can be involved in their antioxidative potential, what is in agreement with our previous observation [11]. Bactericidal effect of AB2 dye against *Staphylococcus aureus* may result from the phenazine structure of dye, as well as from the presence of electron-donating groups. Good dyeing properties of tested AB2 dye may be the result of ionic bonds formation between the free amino groups of wool fibre and carboxyl groups of the dye (Figure 8a), what is in agreement with good dyeing ability of other dyes obtained from laccase-mediated oxidation of carboxylic acid as well as natural dyes [21,27].

This finding could shed new light on the potential uses of laccase in biocatalysis, especially in the eco-friendly synthesis of new bioactive compounds. The combination of the dyeing properties with antioxidative and antimicrobial activity of phenazines is very desirable in the textile industry. It prevents both fading of textiles and colonization of bacteria; therefore, it can be used for medical purposes, especially in hospitals with strict sanitary requirements as well as by immunocompromised patients [31].

## 4. Materials and Methods

### 4.1. Catalyst

The white rot fungus *Cerrena unicolor* (collection number FCL139, obtained from the Fungal Collection of the Biochemistry and Biotechnology Department of Maria Curie-Skłodowska University, Lublin, Poland) was the source of the extracellular laccase (LAC). LAC was obtained and purified using a previously described procedure [32]. LAC activity was determined using 2,2′-azino-bis(3-ethylbenzthiazoline-6-sulphonic acid (ABTS) as a substrate [33]. The reaction mixture contained 50 µL of a LAC sample and 200 µL of ABTS (2.5 mM final concentration) suspended in 750 µL of 0.1 M sodium-tartrate buffer pH 3. Oxidation of ABTS was monitored spectrophotometrically for one minute at 414 nm (λ_414_ = 36,048 M^−1^ cm^−1^). The activity of LAC was expressed in U/mL, where one unit (U) of the enzyme oxidised 1 µmol of ABTS per 1 min at 25 °C.

### 4.2. Characteristics of Substrates

Chemicals, i.e., tartaric acid, 2-amino-3-methoxybenzenesulfonic acid (substrate **A**), ABTS and 5-aminonaphthalenesulfonic acid (5ANS, **B1**) were purchased from Sigma-Aldrich (St. Louis, MO, USA), 2-aminonaphthalenesulfonic acid (2ANS, **B2**) and 4-aminonaphthalenesulfonic acid (4ANS, **B3**) from Aldrich (Buchs, Switzerland and Munich, Germany, respectively). All chemicals were of analytical grade and were used without further purification. The kinetic constants *K*_M_ of the aromatic substrates were monitored at pH 4.5 of 0.1 M sodium-tartrate buffer for each tested compound and fitted directly to the Michaelis–Menten equation (OriginLab software, Northhampton, MS, USA).

Oxygen uptake of the substrates was monitored during their homomolecular transformation by LAC with a biological oxygen monitor (YSI model 5300). The standard vessel contained 3 mL of the transformation mixture: 1 mM concentration of the substrate in 0.1 M sodium-tartrate buffer with pH 4.5. Each measurement was carried out for 3 min of the transformation and the oxygen uptake was calculated in nmol O_2_/mL/min according to Bernhardt et al. [34].

Cyclic voltammetry measurement of the substrates was carried out with a µAUTOLAB type III potentiostat/galvanostat (Metrohm Autolab B.V., Utrecht, Netherlands) using a three-electrode cell containing a saturated Ag/AgCl/KCl_sat_ reference electrode, a platinum wire counter electrode, and a 2-mm diameter GC working electrode (all purchased from MTM-Anko, Kraków, Poland). The potential was scanned from −400 to 1500 mV vs. Ag/AgCl/KCl_sat_ after holding the electrochemical system at the initial potential for 10 s and at the scan speed of 50 mV/s. All measurements were done for 1 mM substrates in 0.1 M sodium tartrate buffer at pH 4.5 in triplicate at room temperature. The measured potentials recorded vs. the Ag/AgCl/KCl_sat_ electrode were corrected by +0.199 V to the normal hydrogen electrode (NHE).

### 4.3. Heteromolecular Transformation of Substrates

1-mL transformation mixtures containing an equimolar concentration of substrates A and B (B1, B2 or B3) with the final concentration of 1 mM were transformed in buffered conditions (0.1 M sodium-tartrate buffer) with different values of pH (range from 3 to 5.5) using LAC with 1 U/mL final activity. The transformation of the substrates was monitored spectrophotometrically at a wavelength characteristic for each product for one minute and expressed as absorbance per minute. Additionally, the UV-Vis spectrum was recorded after the transformation of the substrates. At optimal values of pH during heteromolecular transformations, the substrates were oxidised by LAC in different molar ratios, wherein the maximal concentration of substrate A did not exceed 1 mM. Moreover, the transformations of the substrates were also performed in the presence of 10% of organic solvents such as methanol, acetonitrile, and ethyl acetate to find their influence on the formation of the main product and to improve the solubility of the precursors.

### 4.4. Large-Scale Study of AB2 Dye Synthesis

Three different concentrations of the mixtures of substrates A and B2 (1, 5, and 10 g/L) at an equimolar ratio were oxidised by LAC with different activity ranging from 12.5 U/g to 500 U/g in 100 mM tartrate buffer pH 4. The transformation mixtures were analysed using the HPLC method after 24 and 48 h to assess the optimal LAC activity during the first hours of substrate oxidation. Simultaneously, the absorbance of all the coloured products was monitored at 500 nm using a plate reader (Spark, Tecan, Grödig, Austria).

The oxidation of 5 g/L of the A and B2 substrate mixture (equimolar ratio) using the optimal LAC activity (200 U/g) and 100 mM tartrate buffer pH 4 was monitored for 5 days. At a specified time of oxidation (2, 5, 24, 29, 48, 72, 96, and 120 h), 1 mL of the transformation mixture containing 5 mg of the substrate mixture was centrifuged at 13,000 rpm for 15 min. The supernatant contained dissolved substrates and a fraction of the AB2 dye dissolved in water/buffer medium. The precipitate containing an insoluble AB2 product was dissolved in methanol (percentage up to 90%). Both fractions, i.e., the precipitate and the supernatant, were analysed using HPLC to evaluate the increasing amount of the main product (AB2 dye) and the rate of transformation of substrates A and B2, respectively. Simultaneously, the absorbance of all the coloured products was monitored spectrophotometrically at 500 nm using a plate reader (Spark, Tecan, Grödig, Austria).

### 4.5. HPLC Analyses

High performance chromatographic analyses were performed using an Agilent 1260 Infinity chromatograph coupled with a DAD detector. The biotransformation of an equimolar ratio of substrates A and B2 was monitored by reverse-phase C18 HPLC using a Phenomenex Kinetex Polar C18 (4.6 × 100 mm, 2.6 µm) column. The isocratic elution was prepared using methanol (eluent A) and 50 mM formate buffer (eluent B) in the ratio of 45% of eluent A (*v*/*v*). The pH of eluent B was adjusted to 4.1 using 1 M NaOH. The eluent flow rate was set at 1 mL min^−1^ and the separation column was maintained at 40 °C. Each 3-µL sample was injected using an autosampler. The total run time of each analysis was 20 min. The elution of compounds was monitored at a wavelength of 280 and 500 nm. Agilent Open Lab software (Agilent OpenLab CDS Chem Station product version A.02.10 [026]) was used for data processing and reporting. Identification of substrate peaks was achieved by comparing retention times with the standards.

### 4.6. General Procedure of Dye Synthesis in Large Volume

Preparative scale reactions were performed in a 500-mL Erlenmeyer flask containing the buffered mixture of an equimolar ratio of substrate A and substrate B1, B2, or B3 using the same concentration of each compound. The transformation mixture was prepared as follows: precursors dissolved in water with the addition of 1 M NaOH (3 mM final concentration of each substrate) were added to the sodium-tartrate buffer solution (5–10 mM final tartaric acid concentration) and then the pH value of the mixture was adjusted to the optimal pH value in the range from 4 to 4.5 using 1 M NaOH. The transformation of the substrates into phenazines was carried out on a rotary shaker (120 rpm) at 28 °C using LAC with 1 U/mL final activity.

### 4.7. General Procedure for Purification of the AB1 and AB3 Dyes

At pH 4.5, an orange-red solution of the AB1 and AB3 compounds were obtained and lyophilised. The residues were dissolved with methanol. The products were analysed using MERCK HPTLC Silicagel 60 plates and purified using preparative silica gel column chromatography: FLUKA Silicagel 60 A (200 micron) and a Flash-HPLC system (Gilson, France) (mobile phase: ethyl acetate-MeOH-water: 3:2:1, *v*/*v*) to obtain three fractions for each dye. For both tested dyes, the last red fraction eluted characterised by high intensity of colour was dried under vacuum to obtain the main product of the AB1 and AB3 compounds, i.e., **AB1**: 10-((2-carboxy-6-methoxyphenyl)amino)-11-methoxy-4-sulfobenzo [a]phenazine-8- carboxylic acid (AB1): red solid; UV-Vis: 495 nm; ES-MS: 548 [M-H]^−^. **AB3**: 10-((2-carboxy-6-methoxyphenyl)amino)-11-methoxy-5-sulfobenzo [a]phenazine-8-carboxylic acid (AB3): red solid soluble in water; UV-Vis: 505 nm; ES-MS: 548 [M-H]^−^ and 550 [M+H]^+^, 572 [M+Na]^+^, 1121 [2M+Na]^+^.

### 4.8. General Procedure for Purification of Dye AB2

At pH 4, an orange/red solution of the AB2 compound was obtained and lyophilised. The residue was dissolved with methanol. The products were analysed using MERCK HPTLC Silicagel 60 plates and purified using preparative silica gel column chromatography: FLUKA Silicagel 60 A (200 micron) and a Flash-HPLC system (Gilson) (mobile phase: ethyl acetate-MeOH-water: 3:2:1, *v*/*v*) to obtain four fractions. The last eluted red fraction was dried under vacuum to obtain the AB2 compound, which was identified as a mixture of two compounds: the main product AB2a and AB2b with different mass spectra. **AB2**: 10-((2-carboxy-6-methoxyphenyl)amino)-11-methoxybenzo[a]phenazine-8-carboxylic acid: red solid insoluble in water, soluble in 50% methanol; UV-Vis: 495 nm; ES-MS: 470 [M+H]^+^, 456 [M+H]^+^.

### 4.9. Molecular Characterisation of Products

Low-resolution mass spectroscopy analyses were recorded by an LC/MSD chromatography system (1100 Agilent, Waldbronn, Germany) connected to a UV detector. The analyses were performed in a mixture of MeOH/H_2_O 95:5 at 0.4 mL min^−1^ by direct injection in Electrospray Ionization (*N*_2_ = flow 9 L/min, *T* = 350 °C, solubilisation *p* = 40 PSI, potential difference = 70 eV). NMR spectra were performed at 14.1 T with a Bruker Avance 600 MHz spectrometer at controlled temperatures (25 °C). The solvent is specified for each spectrum. Splitting patterns are designated as s, singlet; d, doublet; t, triplet; q, quartet; m, multiplet; br, broad. Chemical shifts (d) are given in ppm relative to the resonance of their respective residual solvent peak. FTIR spectra were obtained with an ATRFTIR spectrophotometer (Agilent Technologies Cary 630).

### 4.10. Bioactive Properties of Products

#### 4.10.1. Chemiluminescence Assay of Antioxidative Activity

The antioxidant properties of the dyes were assayed using a chemiluminescence method described by Cheng and co-workers [35] based on the measurement of luminescence emitted by the Fe^2+^-EDTA-H_2_O_2_-luminol system. The reaction mixture contained 50 mM phosphate buffer (pH 7.4), 2 mM luminol in 95% ethanol, 1.5 mM Fe^2+^-EDTA solution, and 4.4 mM H_2_O_2_. Samples of the tested dyes (100 µL) at concentrations ranging from 0.05 to 2.5 mg/mL were added to 600 µL of phosphate buffer and mixed with 100 µL of luminol. In the next step, the samples were placed in the luminometer and 100 μL of FeSO_4_ and 100 μL of H_2_O_2_ were added using automatic luminometer dispensers. Standards (vitamin C and trolox) that are well known for their strong antioxidant activity were used as positive controls. The chemiluminescence peak signals were recorded in the absence (*I_o_*) or presence (*I_1_*) of the tested compounds using a Lumat LB 9506 luminometer (Berthold, Germany) with two dispensers. The inhibitory rate (*I_R_*) was calculated according to the following equation:

(1) $$I_{R}=\left(1-\frac{I_{1}}{I_{O}}\right)\times 100\%$$

The EC_50_ values of the dyes were calculated using OriginLab software (Northhampton, MS, USA).

#### 4.10.2. Agar Diffusion Test and Suspension Culture

The qualitative antibacterial properties of all synthesised compounds (AB1, AB2, AB3) were analysed using an agar diffusion test against reference strains of Gram-positive and Gram-negative bacteria (*Staphylococcus aureus* ATCC 25923 and *Escherichia coli* ATCC 25922, respectively). Bacterial *inoculum* was prepared by adjusting the turbidity of each culture of the bacterium to reach an optical density not exceeding the 0.5 McFarland standard, corresponding to approximately 1–4 × 10^8^ colony-forming units per millilitre (CFU/mL). The inoculates were diluted in sterile distilled water. Petri dishes (90 mm diameter) containing Mueller-Hinton broth medium (M-H) agar were inoculated with 100 µL of the bacterial *inoculum*. 100 µL of the tested compound (concentration of 10 mg/mL) were placed into the 9 mm diameter hole in the centre of the Petri dish. Next, the bacteria were cultivated for 24 h at 37 °C. For phenazine AB2, which exhibited the highest antimicrobial effect against *S. aureus*, the quantitative analyses were carried out to obtain the minimal inhibitory concentration (MIC) and the minimum bactericidal concentration (MBC). The tests were performed in a mixture of 200 µL of the M-H medium and the purified AB2 dye with concentrations in a range from 0.010 to 4 mg/mL inoculated with 10 µL of the bacterial culture. The bacteria were cultivated for 24 h at 37 °C and the cultures were analysed after this time. The lack of turbidity of the bacterial culture indicated the value of the MIC parameter. To obtain the value of the MBC parameter, 10 µL of the culture were placed into the sterile M-H medium and cultivated for 24 h at 37 °C. The lack of turbidity of the bacterial culture indicated the value of the MBC parameter.

### 4.11. Wool Dyeing

Wool fabrics for SEM analysis were dyed with the AB2 compound in a bath at a concentration of 5 and 10% relative to the fibre mass (5 or 10% dye applied omf) and in the presence of 1% acetic acid. The bath temperature was 100 °C and the dyeing process lasted 30 min. Afterwards, wool samples were prepared for analysis of the growth inhibition effect against *S. aureus*. Cultures in the mid-exponential growth phase were diluted with LB medium to the cell density of 10^2^ CFU and cultured in the presence of dyed and non-dyed wool fibres overnight. Next day, 20 µL of 10,000- and 25,000-times diluted bacterial cultures were inoculated on Petri dishes and cultivated for 24 h. Afterwards, the bacterial cells were counted to obtain the inhibition of bacterial growth (%) in contrast to the control culture without addition of dyed wool. Wool fabrics for colour fastness test were dyed using 1% solution of AB2 dye and analysed by the TKANLAB Laboratory (Łódź, Poland). The characteristic of wool fabrics: yarn linear mass-warp R63 ± 4/2 weft R74 ± 4/2; number of threads per 10 cm—warp 175 ± 10, weft 135 ± 8; mass per unit area—215 ± 10 g/m^2^; fat content—0.8 ± 0.3%; linen weave.

### 4.12. SEM Analysis

Wool fibres stained with the AB2 dye were fixed in 3% glutaraldehyde in phosphate buffer (PBS pH 7.4) for 15 min at room temperature. After rinsing several times with 0.1 M PBS, the wool fibres were dehydrated in increasing concentrations of ethanol (25, 60, 90, and 100%) for 5 min in every solution. After rinsing out the alcohol, the samples were dried overnight in a desiccator in the presence of calcium chloride. Finally, the samples were coated with gold in an Emitech K550X Sputter Coater and observed with a TESCAN Vega 3 LMU microscope (Brno-Kohoutovice, Czech Republic).

## 5. Conclusions

In this work, the formation of novel bioactive phenazine compounds in the one-step sustainable and environmentally friendly enzymatic synthesis was described. The known chemical route of phenazine synthesis requires the use of coupling agents and additives that are very often toxic and mutagenic. The proposed laccase-mediated bioconversion of benzoic and naphthalene amino derivatives into bioactive dyes with the phenazine structure showed attractive advantages, including the mild reaction conditions and the short reaction time, compared to the established chemical oxidative methods. The structure and properties of the novel dyes were characterised using a variety of analytical and spectroscopic techniques. During specific tests, one new dye with the tri-molecular phenazine structure and antibacterial activity against *Staphylococcus aureus* was found. The proposed enzymatic route is a valuable and promising approach for the synthesis of different phenazine dyes with antimicrobial and antioxidative activity.

## Figures and Tables

**Table 1:** Electrochemical and kinetic parameters of tested substrates.

Substrates Acronyms	Oxygen Uptake ^1^ (nmol O_2_/min/mL)	*E*_pa_^1^ (V)	λ (nm)	*K*_M_ (mM)
A	352.29	0.805	490	1.58
B1	134.98	0.879	460	3.94
B2	23.31	1.021	495	17.00
B3	34.24	0.913	495	5.38

^1^ The determination of oxygen uptake and oxidation potential (vs. NHE) were performed at the Na-tartrate buffer pH 4.5 value for 1 mM concentration of each substrate.

**Table 2:** Heteromolecular coupling reactions between substrate I (substrate A) and substrate II (substrate B1, B2 or B3), mediated by fungal LAC.

Substrate I	Substrate II	Reaction pH Value	Isolated Yields (%)
A	B1	4.5	19
A	B2	4	25
A	B3	4.5	27

**Table 3:** Antioxidative properties of the phenazine dyes tested by chemiluminescence assay and expressed as EC_50_ values in comparison to the vitamin C and trolox as positive controls.

Tested Compound	EC_50_ (mg/mL)
AB1	0.47
AB2	0.44
AB3	0.63
vitamin C	0.0047
trolox	0.00033

**Table 4:** Colour fastness of wool fabric dyed with the AB2 dye at a concentration of 1%.

Tested Parameters of Colour Fastness		Scale	ISO Standard
Artificial light ^1)^	a)	5	PN-EN ISO 105-B02:2014-11
Distilled water ^2)^	a)	4	PN-EN ISO 105-E01:2013-06
	b)	4–5	
	c)	3–4	
Washing 40 °C ^2)^	a)	3–4	PN-EN ISO 105-C06:2010
	b)	4–5	
	c)	4–5	
Alkaline sweat ^2)^	a)	4	PN-EN ISO 105-E04:2013-06
	b)	3–4	
	c)	2–3	
Acidic sweat ^2)^	a)	4	PN-EN ISO 105-E01:2013-06
	b)	4	
	c)	3	
Dry rubbing ^2)^	b)	4–5	PN-EN ISO 105-X12:2005
Wet rubbing ^2)^	b)	4–5	PN-EN ISO 105-X12:2005

Colour fastness according to a blue ^1)^ or grey ^2)^ scale, in which index “8” or “5”, respectively, means no change of colour and “1” means great change in colour; according to PN-EN20105-A02:1996 and PN-EN 20105-A03:1996 standards; a) change in the colour of the tested sample; b) soiled whiteness of the accompanying fabric—cotton; c) soiled whiteness of the accompanying fabric—wool.

## Supplementary Materials

Supplementary Materials can be found at https://www.mdpi.com/1422-0067/21/6/2052/s1, Table S1. NMR and FTIR data.

## Abbreviations

LAC	Laccase
omf	on mass fibre
MIC	Minimal Inhibitory Concentration
SEM	Scanning Electron Microscope

## References

[1] Rodriguez Couto S., Toca Herrera J.L. (2006). Industrial and biotechnological applications of laccases: A review. Biotechnol. Adv..

[2] Mate D.M., Alcalde M. (2016). Laccase: A multi-purpose biocatalyst at the forefront of biotechnology. Microb. Biotechnol..

[3] Mikolasch A., Schauer F. (2009). Fungal laccase as tools for the synthesis of new hybrid molecules and biomaterials. App. Microbiol. Biotechnol..

[4] Blankenfeldt W., Parsons J.F. (2014). The structural biology of phenazine biosynthesis. Curr. Opin. Struc. Biol..

[5] Laursen J.B., Nielsen J. (2004). Phenazine natural products:  biosynthesis, synthetic analogues, and biological activity. Chem. Rev..

[6] Hayden C., Bryant J.J., Mackey M.A., Höfer K., Lindner B.D., Nguyen V.P., Jäschke A., Bunz U.H. (2014). Antimicrobial activity of water-soluble triazole phenazine clickamers against *E coli*. Chem. Eur. J..

[7] Guttenberger N., Blankenfeldt W., Breinbauer R. (2017). Recent developments in the isolation, biological function, biosynthesis, and synthesis of phenazine natural products. Bioorg. Med. Chem..

[8] Krishnaiah M., Rodrigues de Almeida N., Udumula V., Song Z., Chhonker Y.S., Abdelmoaty M.M., Aragao do Nascimento V., Murry D.J., Conda-Sheridan M. (2018). Synthesis, biological evaluation, and metabolic stability of phenazine derivatives as antibacterial agents. Eur. J. Med. Chem..

[9] Smitka T.A., Bunge R.H., Wilton J.H., Hokanson G.C., French J.C. (1986). PD 116,152, a new phenazine antitumor antibiotic. Structure and antitumor activity. J. Antibiot. (Tokyo).

[10] Mahran A.M., Ragab S.S., Hashem A.I., Ali M.M., Nada A.A. (2015). Synthesis and antiproliferative activity of novel polynuclear heterocyclic compounds derived from 2,3-diaminophenazine. Eur. J. Med. Chem..

[11] Polak J., Jarosz-Wilkołazka A., Szałapata K., Grąz M., Osińska-Jaroszuk M. (2016). Laccase-mediated synthesis of a phenoxazine compound with antioxidative and dyeing properties–the optimisation process. New Biotechnol..

[12] Alonso M., Horcajada R., Groombridge H.J., Chudasama (née Mandalia) R., Motevalli M., Utley J.H., Wyatt P.B. (2005). Synthesis of phenazine derivatives for use as precursors to electrochemically generated bases. Org. Biomol. Chem..

[13] Forte S., Polak J., Valensin D., Taddei M., Basosi R., Vanhulle S., Jarosz-Wilkołazka A., Pogni R. (2010). Synthesis and structural characterization of a novel phenoxazinone dye by use of a fungal laccase. J. Mol. Catal. B Enzym..

[14] Bruyneel F., Enaud E., Billottet L., Vanhulle S., Marchand-Bryneart J. (2008). Regioselective synthesis of 3-hydroxyorthanilic acid and its biotransformation into a novel phenoxazinone dye by use of laccase. Eur. J. Org. Chem..

[15] Sousa A.C., Conceição Oliveira M., Martins L.O., Robalo M.P. (2014). Towards the rational biosynthesis of substituted phenazines and phenoxazinones by laccases. Green Chem..

[16] Sousa A.C., Oliveira M.C., Martins L.O., Robalo M.P. (2018). A sustainable synthesis of asymmetric phenazines and phenoxazinones mediated by CotA-laccase. Adv. Synth. Catal..

[17] Hahn V., Davids T., Lalk M., Schauer F., Mikolasch A. (2010). Enzymatic cyclizations using laccases: Multiple bond formation between dihydroxybenzoic acid derivatives and aromatic amines. Green Chem..

[18] Singh R., Jain A., Panwar S., Gupta D., Khare S.K. (2005). Antimicrobial activity of some natural dyes. Dyes Pigm..

[19] Widsten P., Healthcote C., Kandelbauer A., Guebitz G., Nyanhongo G.S., Prasetyo E.N., Kudanga T. (2010). Enzymatic surface functionalisation of lignocellulosic materials with tannins for enhancing antibacterial properties. Process Biochem..

[20] Jeon J.-R., Baldrian P., Murugesen K., Chang Y.-S. (2012). Laccase-catalysed oxidations of naturally occurring phenols: From in vivo biosynthetic pathways to green synthetic applications. Microb. Biotechnol..

[21] Ghaheh F.S., Mortazavi S.M., Alihosseini F., Fassihi A., Nateri A.S., Abedi D. (2014). Assessment of antibacterial activity of wool fabrics dyed with natural dyes. J. Clean. Prod..

[22] Mikolasch A., Wurster M., Lalk M., Witt S., Seefeldt S., Hammer E., Schauer F., Jülich W.D., Lindequist U. (2008). Novel beta-lactam antibiotics synthesized by amination of catechols using fungal laccase. Chem. Pharm. Bull..

[23] Mikolasch A., Hildebrandt O., Schlüter R., Hammer E., Witt S., Lindequist U. (2016). Targeted synthesis of novel β-lactam antibiotics by laccase-catalyzed reaction of aromatic substrates selected by pre-testing for their antimicrobial and cytotoxic activity. Appl. Microbiol. Biotechnol..

[24] Polak J., Jarosz-Wilkolazka A., Szuster-Ciesielska A., Wlizlo K., Kopycinska M., Sojka-Ledakowicz J., Lichawska-Olczyk J. (2016). Toxicity and dyeing properties of dyes obtained through laccase-mediated synthesis. J. Clean. Prod..

[25] Polak J., Jarosz-Wilkołazka A. (2012). Structure/redox potential relationship of simple organic compounds as potential precursors of dyes for laccase-mediated transformation. Biotechnol. Progr..

[26] Polak J., Jarosz-Wilkolazka A. (2012). Fungal laccases as green catalysts for dye synthesis. Process Biochem..

[27] Wlizło K., Polak J., Jarosz-Wilkolazka A., Pogni R., Petricci E. (2020). Novel textile dye obtained through transformation of 2-amino-3-methoxybenzoic acid by free and immobilised laccase from a *Pleurotus ostreatus* strain. Enz. Microb. Technol..

[28] Sousa A.C., Piedade M.F.M., Martins L.O., Robalo M.P. (2016). Eco-friendly synthesis of indo dyes mediated by a bacterial laccase. Green Chem..

[29] Xu F. (1997). Effects of redox potential and hydroxide inhibition on the pH activity profile of fungal laccases. J. Biol. Chem..

[30] Shleev S., Klis M., Wang Y., Rogalski J., Bilewicz R., Gorton L. (2007). Comparative spectroelectrochemical studies of lyophilized and nonlyophilized laccases from *Cerrena unicolor* basidiomycete. Electroanalysis.

[31] Shahid M., Mohammad F., Chen G., Tang R.C., Xing T. (2016). Enzymatic processing of natural fibers: White biotechnology for sustainable development. Green Chem..

[32] Luterek J., Gianfreda L., Wojtaś-Wasilewska M., Rogalski J., Jaszek M., Malarczyk E., Dawidowicz A., Ginalska G., Leonowicz A., Finks-Boots M. (1997). Screening of the wood rotting fungi for laccase production: Induction by ferulic acid, partial purification, and immobilization of laccase from the high laccase-producing strain, *Cerrena unicolor*. Acta Microbiol. Pol..

[33] Wolfenden B.S., Willson R.L. (1982). Radical-cations as reference chromogens in kinetic studies of ono-electron transfer reactions: Pulse radiolysis studies of 2,2’-azinobis-(3-ethylbenzthiazoline-6-sulphonate). J. Chem. Soc. Perkin. Trans..

[34] Bernhardt F.-H., Staudinger H., Ulrich V. (1970). The properties of p-anisate O-demethylase in cell-free extracts of *Pseudomonas* sp.. Hoppe-Seyler’s Z. Physiol. Chem..

[35] Cheng Z., Yan G., Li Y., Chang W. (2003). Determination of antioxidant activity of phenolic antioxidants in a Fenton-type reaction system by chemiluminescence assay. Anal. Bioanal. Chem..

