# Supplementary material for: Structure and Bioactive Properties of Novel Textile Dyes Synthesised by Fungal Laccase
Source: Int J Mol Sci. 2020 Mar 17;21(6):2052. doi: 10.3390/ijms21062052 (PMC7139866; doi:10.3390/ijms21062052)
Supplement: Supplementary file 1 [file ijms-21-02052-s001.pdf]

Supplementary material

Table S1. NMR and FTIR data.

Dye	NMR data	FTIR data
AB1	1H NMR (600 MHz, DMSO) δ 9.35 (d, J = 6.3 Hz, 1H), 9.09 (d, J = 6.2 Hz, 1H), 8.27 (d, J = 6.2 Hz, 1H), 8.18-8.09 (m, 2H), 7.90 - 7.69 (m, 4H), 7.20 (d, J = 6.9 Hz, 1H), 7.01 - 6.95 (m, 2H), 6.43 (bs, 1H), 3.95 (s, 3H), 3.70 (s, 3H). 13C NMR (600 MHz, DMSO-d6/H2O) δ 172.57 (CO), 170.16 (CO), 154.27 (C), 145.70 (2 C), 144.79 (C), 138.59 (C), 130.79 (CH), 128.73 (3 C), 127.97 (2 CH), 126.77 (C), 125.67 (2 CH), 124.30 (CH), 123.29 (CH), 122.64 (3 CH),115.57 (CH), 56.92 (2 OCH_3).	FTIR ν(cm^{-1}): 3361, 3277, 3231, 3185, 2925, 2854, 2130, 1944, 1567, 1490, 1435, 1381, 1338, 1297, 1230, 1198, 1165, 1050, 1025, 914, 894, 804.
AB2	1H NMR (600 MHz, DMSO-d6) δ 8.28 (d, J = 6.4 Hz, 1H), 8.15-8.17 (m, 1H), 8.08 (t, J = 6.1 Hz, 1H), 8.04 – 7.90 (m, 2H), 7.72-7.73 (m, 1H), 7.21 – 7.55 (m, 3H), 7.11 (d, J = 7.3 Hz, 1H), 6.91 (bs, 2H), 6.52 (bs, 1H), 3.80 (s, 6H).; 13C NMR (600 MHz, DMSO-d6/H_2O) δ 178.55 (CO), 175.31 (CO), 153.74 (C), 153.54 (C), 144.96 (2 C), 139.48 (C), 134.74 (C), 131.99 (3 C), 130.05 (2 CH), 128.51 (2 CH), 128.30 (CH), 127.80 (2 C), 127.47 (C), 127.26 (CH), 125.65 (2 CH), 124.45 (2 CH),119.98 (C), 56.84 (OCH_3), 48.92 (OCH_3).	FTIR ν(cm^{-1}): 3361, 2922, 2850, 2367, 2355, 2140, 1650, 1559, 1400, 1381, 1282, 1242, 1223, 1178, 1053, 1013, 925, 825.
AB3	1H NMR (600 MHz, DMSO) δ 9.29 (d, J = 7.2 Hz, 1H) 8.94 (d, J = 7.8 Hz, 1H), 7.97-7.86 (m, 4H), 7.71 (s, 1H), 7.58 (d, J = 10.2 Hz, 1H), 7.34 (bs, 2H), 6.40 (s, 1H), 3.98 (s, 3H), 3.67 (s, 3H); 13C NMR (600 MHz, DMSO-d6/H_2O) δ 172.32 (CO), 170.21 (CO), 146.52 (C), 145.11 (2C), 138.95 (2C), 129.21 (2CH), 128.77 (2C), 128.69 (CH), 128.41 (2C), 127.23 (2C), 126.97 (CH), 124.72 (CH), 123.71 (C), 121,36 (CH), 114.74 (CH), 108.79 (C), 107.48 (CH), 98.33 (CH), 57.21 (2 OCH_3).	FTIR ν(cm^{-1}): 3307, 2974, 2871, 2375, 2355, 2133, 1559, 1476, 1428, 1370, 1336, 1281, 1220, 1213, 1140, 1046, 1034, 1010, 967, 911, 903, 810.
